# Leukocyte-derived ratios are associated with late-life any type dementia: a cross-sectional analysis of the Mugello study

**DOI:** 10.1007/s11357-021-00474-3

**Published:** 2021-10-21

**Authors:** Gemma Lombardi, Roberto Paganelli, Michele Abate, Alex Ireland, Raffaele Molino-Lova, Sandro Sorbi, Claudio Macchi, Raffaello Pellegrino, Angelo Di Iorio, Francesca Cecchi

**Affiliations:** 1 IRCCS Fondazione Don Carlo Gnocchi, Via di Scandicci 269, 50143 Florence, Italy; 2 Department of Neuroscience, Psychology, Drug Research and Child Health (NEUROFARBA), University of Florence, Florence, Italy; 3 Department of Medicine and Science of Aging, Laboratory of Clinical Epidemiology and Aging, University Centre of Sports Medicine, University “G. d’Annunzio, Viale Abruzzo 322, Chieti, Italy; 4 Institute of Clinical Immunotherapy and Advanced Biological Treatments, YDA, Pescara, Italy; 5 Department of Life Sciences, Musculoskeletal Science and Sports Medicine Research Centre, Manchester Metropolitan University, John Dalton Building, Chester Street, Manchester, M1 5GD UK; 6 Department of Experimental and Clinical Medicine, Università Di Firenze, Largo Brambilla 3, 50100 Florence, Italy

**Keywords:** Inflamm-aging, Immunosenescence, Neuroinflammation, Lymphocyte count, Lymphocyte-to-monocyte-ratio, Dementia

## Abstract

Immunosenescence, vascular aging, and brain aging, all characterized by elevated levels of inflammatory markers, are thought to share a common pathogenetic pathway: inflamm-aging. Retrospective cross-sectional analysis was conducted using data from the Mugello study (Tuscany, Italy), a representative Italian cohort of free-living nonagenarians. to assess the association between specific peripheral inflammation markers derived from white blood cell counts, and the diagnosis of dementia. All the variables of interest were reported for 411 subjects (110 males and 301 females) out of 475 enrolled in the study. Anamnestic dementia diagnosis was obtained from clinical certificate and confirmed by a General Practitioner, whereas leukocyte ratios were directly calculated from white blood cell counts. Body mass index and comorbidities were considered potential confounders. Diagnosis of any type dementia was certified in 73 cases (17.8%). Subjects affected by dementia were older, more frequently reported a previous stroke, had lower body mass index, and lower Mini-Mental-State-Examination score. Moreover, they had a higher lymphocyte count and lymphocyte-to-monocyte ratio compared to the non-demented nonagenarians. We found that higher levels of lymphocyte counts are cross-sectionally associated with a clinical diagnosis of dementia. Furthermore, lymphocyte-to-monocyte ratio is directly associated with any type of dementia, independently of age, sex, lymphocyte count, and comorbidities. Lymphocyte-to-monocyte ratio may be considered a marker of immunological changes in the brain of dementia patients; moreover, it is low-cost, and easily available, thus enabling comparisons among different studies and populations, although the timeline and the extent of lymphocyte-to-monocyte ratio role in dementia development must be further investigated.

## Introduction

Dementia is a chronic, progressive syndrome affecting both cognitive and functional abilities, representing one of the major causes of disability and dependency among older people. The incidence of dementia increases exponentially with age, before the age of 90 years, but few data are available for the oldest-old and for centenarians [1]. The aging process is associated with chronic low-grade inflammation, that is present not only in peripheral tissues, but involves also the brain [2]. Converging evidence supports the hypothesis that both central and peripheral inflammation play a pivotal role in the pathogenesis of dementia [3]. The central nervous system (CNS) inflammation process has polyhedric manifestations, occurring in the continuum of the different stages of dementia [4]; moreover, different forms of dementia might share inflammation as an etiopathological trigger and a common pathway [5]. In older individuals, due to multiple systemic inflammatory events [6], and in the earliest silent dementia phase, in response to intraneuronal accumulation of oligomeric peptides, neurotoxic cytokines are released [7]: in this inflammatory scenario, microglia are activated [8], driving further proinflammatory conditions which in turn induce an anti-inflammatory response [9]. The progression of these processes culminates with the imbalance between adaptive/innate immune response, CNS invasion by peripheral monocytes [10], lymphocyte proliferation [7], and hyperactivation of microglial phagocytic cells [2]. Because of the association between dementia pathogenesis and the immune system, several studies on immune cells as biological markers are emerging [11]. The recent COVID-19 pandemic has renewed the interest in leukocyte count-derived indexes, which are easily available at no extra cost, and represent possible markers of inflammation with predictive value for disease outcome, not only in the elderly. The leukocytes derived ratio, as a combined inflammatory biomarker, integrates information from innate and adaptive immunity [5]. It avoids the disadvantage of an absolute value of a single leukocyte subtype, which may be affected by infection or dehydration, and has higher clinical significance than the other independent inflammatory biomarkers [12]. In the context of dementia, one case–control study has been published, reporting that leukocyte ratios can discriminate patients with dementia from controls [13]. In a free living “young-old” population (mean age 61 years), the Rotterdam study reported an increase of Neutrophils and contextually a decrease of lymphocyte absolute numbers, with dementia patients showing a higher activation of the innate immune system, measured by different leukocytes ratios, compared to controls [14]. To date, these associations have not been explored within a representative oldest-old population sample.

The aim of the present study is to evaluate the association of routinely available, specific peripheral inflammation markers, with a diagnosis of dementia, in a cohort of community-dwelling oldest-old participants.

## Materials and methods

The design of the Mugello study has been described in detail elsewhere [15]. Briefly, the project was designed and conducted by the Department of Experimental and Clinical Medicine, University of Florence, Italy, and by the Don Carlo Gnocchi Foundation (Florence, Italy). The study was designed as a cross-sectional survey of subjects aged 90 years and over, living in the Mugello area, a valley spread north-east of Florence, in Tuscany, and data were collected in 2009.

### Samples

In this retrospective study, 475 nonagenarians (130 men and 345 women, age range 90–105 years) were enrolled, representing approximately 65% of those nonagenerians living in the Mugello area; 411 participants who had all the variables of interest were included in this analysis. There were no exclusion criteria. The study protocol, which complied with the principles of the Declaration of Helsinki on clinical research involving human subjects, was approved by the Institutional Review Board. All participants, or their proxies, signed the informed consent form to participate to the study.

### Home interview and diagnoses of pathological conditions

A trained interviewer investigated family, medical, and medication history. Past and recent medical history was recorded using a semi-structured questionnaire [16].

### Comprehensive geriatric assessment

Specific geriatric items, such as functional independence, physical activity level, quality of life, global cognition, mood, sleep quality, and falls, were addressed. The interview was followed by a general physical and clinical examination.

### Blood collection

After the clinical assessment, a nurse collected fasting blood samples for routine and other selected laboratory tests. Serum and plasma aliquots were also stored at − 80° for future investigation.

### Laboratory tests

At the enrollment (2009), several laboratory parameters were assessed. Serum levels of Thyroid Stimulating Hormone (TSH) were measured using an enzyme-linked immunosorbent assay (ELISA), with the WHO First International Reference Standard, and a sensitivity of 0.08 μg/ml. Albumin, and α2-globulin were measured using an agarose gel electrophoresis technique (Hydragel Protein(E) 15/30 and HydraPLUS, Sebia, Issy-les-Moulineaux, France). The C-reactive protein (CRP) assay was performed using an immunoturbidimetric method with a commercial kit (Roche Diagnostics, GmbH, Mannheim, Germany) and a Roche Modular P Chemistry Analyzer (Roche Diagnostics, GmbH, Mannheim, Germany), with a sensitivity of 3 µg/mL; intra-assay coefficient of variability (CVs) for three concentrations were 1.3%, 1.0%, and 0.6%; inter-assay CVs for three concentrations were 6.0%, 2.9%, and 1.3%. Hematological parameters studied were hemoglobin, hematocrit, total white blood cell (WBC), red blood cell (RBC), and platelet (PLT) counts, and the WBC differential count; they were measured using the hematology analyzer Sysmex XT-1800 (Sysmex, Inc., Mundelein, IL). The following leukocyte-derived indexes were evaluated platelet (P)-to-lymphocytes (L) ratio (PLR) [17], the neutrophil (N)-to-lymphocyte ratio (NLR) [18], the lymphocyte-to-monocyte (M) ratio (LMR) [19], and the systemic immune-inflammation index (SII) obtained by multiplying the number of P by that of M, and the product divided by the number of L [20].

Body mass index (*BMI*) was calculated as weight (kg)/height squared (m^2^), and participants were classified as overweight/obese if their *BMI* was above 25.

### Statistical analysis

Continuous variables were reported as mean and standard deviation (SD), whereas dichotomous variables were reported as number and percentages. Participants were divided into two groups according to the presence/absence of a clinical diagnosis of any type of dementia.

Differences between the two groups were evaluated by analysis of variance and chi-square test, for continuous and categorical variables, respectively. Additional analyses with adjustment for age and sex were also reported, since gender dimorphism and age effect could interfere with dependent as with independent variables. To assess the association between any type of dementia with leukocyte-derived indexes, several different logistic regression models were analyzed. Cognitive status (dementia/non-dementia) was assumed as the dependent variable, and the analysis was carried out stratifying for the conditions independently associated with dementia at univariate analysis, adjusting for age, sex, and lymphocyte count. Results were reported as odds ratio (OR) and 95% confidence intervals (95%CI). All statistical analyses were performed using SAS software rel.9.4.

## Results

Four hundred and eleven subjects were included in the study: 301 (73.2%) were female; the mean age (± SD) of the sample was 93.01 ± 3.17 years. Dementia of any type was clinically certified in 73 cases (17.8%). Those subjects were older (94.06 ± 3.66 vs 92.81 ± 3.14 years, *p* value < 0.001), mostly female (79.5%), with lower *BMI* (23.86 ± 4.50 vs 25.41 ± 4.54, *p* value = 0.01) and lower MMSE score (7.05 ± 7.87 vs 21.55 ± 7.91, *p* value < 0.001). In the non-dementia group, on the contrary, albumin concentration was higher (56.52 ± 4.64 vs 54.90 ± 4.59, *p* value = 0.02). C-Reactive Protein, α2-globulin, and TSH level did not differ between the groups (Table 1). Among hematological parameters, lymphocyte count was higher in the dementia-group compared to the non-dementia-group (2.04 ± 1.24 vs 1.75 ± 0.72; *p* value = 0.006); no differences were found in all other markers (RBC, WBC, monocytes, platelets, hemoglobin, and hematocrit), adjusting analysis for age and sex between the two study groups (Table 1). The subjects reporting a clinical diagnosis of dementia showed higher LMR (4.85 ± 3.00 vs 3.83 ± 1.85; *p* value < 0.001) compared to the others. For the other leukocyte-derived ratios, namely, NLR, PLR, and SII, no differences were observed (*p* value = 0.14; *p* value = 0.08; *p* value = 0.14; respectively) (Table 1). Subjects with a diagnosis of dementia showed a higher prevalence of previous stroke (30.1% vs 17.5%, *p*^2^ = 0.007), and a lower prevalence of hypertension (42.5% vs 59.8%, *p*^2^ = 0.003), but no differences in the other major clinical diagnoses considered (Table 2). No multiplicative effect was demonstrated for the interaction of stroke, hypertension, and *BMI* in the association with dementia (data not shown). In the logistic regression model analysis, LMR was directly associated with any type dementia (*OR* = 1.22 95%CI: 1.06–1.42; *p* value = 0.006) independently from age, sex, absolute lymphocyte number, hypertension, stroke, and *BMI*. Interestingly, age and sex did not exert a significant confounding effect on the association, whereas stroke (*p* value = 0.03), hypertension (*p* value = 0.01), and *BMI* (*p* value = 0.04) were independently associated with dementia. Moreover, no significant interaction between dependent variables was found. Therefore, to better define the role of those diseases in the association with dementia, we carried out different stratified-analyses for the three clinical conditions. The associations found are shown in Table 3. In subjects without stroke, LMR was associated with dementia (*OR* = 1.02; 95%CI:1.01–1.03; *p* value = 0.003), but in contrast, the association was no longer present in subjects with a history of stroke (*p* value = 0.38). Obesity (*BMI* > 25) overlapped the trend found for stroke, namely, LMR was associated in subjects with a lower *BMI* (*OR* = 1.04; 95%CI:1.02–1.06; *p* value < 0.001), whereas in overweight/obese-subjects (*BMI* > 25), no association could be found (*p* value = 0.07). The presence or absence of hypertension did not modify the strength of the association between LMR and dementia (*OR* = 1.03;95%CI:1.00–1.05; *p* value = 0.04, and *OR* = 1.02;95%CI:1.01–1.03; *p* value = 0.008, respectively). For these three clinical conditions, no mediation-effect in the association between leukocyte-derived markers and dementia was detected.

**Table 1:** Demographic, anthropometric, and laboratory parameters, reported according to certified clinical diagnosis of any type dementia; *p* value1 = unadjusted *p* value; *p* value2 = sex and age-adjusted *p* value. *BMI*, body mass index; *MMSE*, Mini-Mental-State-Examination; *NLR*, neutrophil-to-lymphocyte ratio; *PLR*, platelet-to-lymphocyte ratio; *LMR*, lymphocyte-to-monocyte ratio; *SII*, systemic immune-inflammation index

Variable	No dementia	Dementia	*p* value1	*p* value2
	338	73		
Age (yy)	92.81 ± 3.14	94.06 ± 3.66	< 0.001	
Sex female	243 (71.9)	58 (79.5)	0.19	
Education (yy)	4.37 ± 2.74	3.86 ± 1.91	0.10	0.42
*BMI*	25.41 ± 4.54	23.86 ± 4.50	0.005	0.01
MMSE score (0–30)	21.55 ± 7.91	7.05 ± 7.87	< 0.001	< 0.001
Neutrophils (× 10^^3^/μl)	3.96 ± 2.49	3.91 ± 1.55	0.87	0.88
Lymphocytes (× 10^^3^/μl)	1.75 ± 0.72	2.04 ± 1.24	0.007	0.006
Monocytes (× 10^^3^/μl)	0.50 ± 0.19	0.45 ± 0.16	0.06	0.12
Platelets (× 10^^3^/μl)	216.02 ± 87.11	215.42 ± 64.70	0.96	0.92
Hemoglobin (g/dl)	12.95 ± 1.54	12.71 ± 1.47	0.22	0.41
Hematocrit (%)	39.10 ± 4.79	37.65 ± 6.82	0.03	0.08
C-reactive protein (mg/dl)	1.016 ± 2.47	1.03 ± 1.76	0.97	0.84
Alpha-2-globulins (%)	12.01 ± 1.84	11.83 ± 2.14	0.45	0.21
Albumin (%)	56.52 ± 4.64	54.90 ± 4.59	0.007	0.02
TSH (μUI/mL)	2.15 ± 5.76	1.99 ± 2.32	0.82	0.65
NLR	2.65 ± 2.65	2.20 ± 1.03	0.15	0.14
PLR	139.80 ± 78.17	122.17 ± 50.24	0.07	0.08
LMR	3.83 ± 1.85	4.85 ± 3.00	< 0.001	< 0.001
SII	570.35 ± 537.38	474.12 ± 272.37	0.14	0.14

**Table 2:** Distribution of main comorbidity diagnosis according to certified clinical diagnosis of any type dementia; *p* value1 = unadjusted *p* value; *p* value2 = sex and age-adjusted *p* value

	No dementia	dementia	*p* value1	*p* value2
	338	73		
Acute myocardial infarction	46 (13.6)	8 (11.0)	0.54	0.89
Congestive heart failure	74 (21.9)	12 (16.4)	0.30	0.34
Peripheral artery disease	60 (17.8)	10 (13.7)	0.40	0.32
Hypertension	202 (59.8)	31 (42.5)	0.007	0.003
Dyslipidemia	38 (11.2)	4 (5.5)	0.19	0.61
Previous Stroke	59 (17.5)	22 (30.1)	0.01	0.007
Pneumonia (anamnestic)	43 (12.7)	12 (16.4)	0.40	0.78
Gastric ulcer	52 (15.4)	7 (9.6)	0.20	0.16
Diabetes	44 (13.0)	12 (16.4)	0.44	0.42
Cancer	46 (13.6)	7 (9.6)	0.35	0.41

**Table 3:** Logistic regression analysis, association between certified clinical diagnosis of any type dementia and lymphocyte-to-monocyte ratio, adjusted for age and sex, stratifying for stroke, hypertension, and obesity (*BMI* > 25)

LMR	No previous stroke	279	51	1.02 (1.01–1.03)	0.003
	Previous stroke	59	22	1.01 (0.99–1.04)	0.38
	No hypertension	136	42	1.03 (1.00–1.05)	0.04
	Hypertension	202	31	1.02 (1.01–1.03)	0.008
	*BMI* < 25	179	52	1.04 (1.02–1.06)	< 0.001
	*BMI* ≥ 25	159	21	1.01 (0.99–1.03)	0.07

**Fig. 1:**
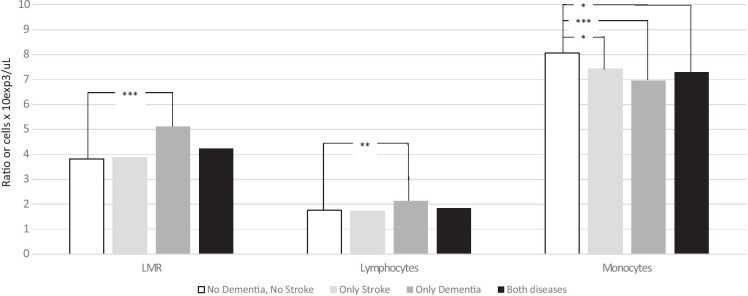
Lymphocyte and monocyte absolute counts, and lymphocyte-to-monocyte ratio (LMR), according to dementia and stroke. *p value < 0.05; **p value < 0.01; ***p value < 0.001

We assessed the differences in the lymphocytes and monocyte absolute counts, and in the LMR, according to dementia and stroke. In subjects reporting only dementia, we found a higher lymphocyte absolute count (*p* value = 0.004), lower monocyte absolute count (*p* value < 0.001), and consequently higher LMR (*p* value < 0.001), compared to subjects who did not have dementia nor stroke. Moreover, in subjects reporting both diseases (dementia and stroke), and in those reporting only stroke, monocyte numbers were lower compared to those who had neither dementia nor stroke (Fig. 1).

## Discussion

In this cross-sectional population study of nonagenarians, representative of the Italian population of this age, we found that a marker of imbalance between innate and adaptive immunity (namely LMR) is directly associated with any type of clinical diagnosis of dementia, independent of age, sex, lymphocyte count, *BMI*, stroke, and hypertension. Furthermore, higher lymphocyte counts are also associated with a clinical diagnosis of dementia, but the strength of the association is reduced and no longer statistically significant when the logistic model includes also the LMR.

Immunosenescence is a decline in the functional efficiency of the immune system, which may be explained in terms of molecular and cellular mechanisms responsible for inflammatory age-associated disorders, and indicated as inflamm-aging [21]. The pathogenetic effect of inflamm-aging can be independent from the total amount of pro-inflammatory mediators, but rather, it could depend on their location, and on the type of activated cells [6]. A hallmark of individuals with dementia is neuroinflammation, an innate immunological response in the nervous system [2]; this has been observed to occur in the brains of those affected [10]. In the immune pathophysiological process of dementia, at least two phases are assumed to exist: the early preclinical stage with a predominantly proinflammatory component, and the late clinical stage, characterized by an imbalance between innate/adaptive systems [2, 4]. Most of the conceptual framework underlying this theoretical pathway was derived from animal models or autopsy of subjects with preclinical symptoms, but without an established diagnosis of dementia [2]. Therefore, much research effort is devoted to identify possible low cost, and highly efficient, biological markers of preclinical dementia, in order to allow a precision medicine approach. In the recent past, the importance of leukocyte counts and their derived ratios has been established [22] in several fields of clinical medicine, as markers of disease progression, predictors of outcomes, and even of cognitive impairment after acute ischemic stroke [23].

In this study, LMR was associated with prevalent diagnosis of dementia, only in subjects without stroke, whereas this association was not present in the group of subjects who had a stroke. In those subjects affected only by dementia, the increase in the LMR was sustained by the lymphocyte absolute count increase, and a contextual monocyte absolute count decrease, clearly showing an imbalance between innate ad adaptive immunity. Conversely, in subjects with a history of stroke, and in those also diagnosed with dementia, only a decrease in monocyte absolute count was observed. In previous studies, LMR was reported to be a marker of clinical progression [24], disease complications and poor outcome [25] in acute ischemic and hemorrhagic stroke, but not in the chronic phase of the disease. The temporal trend of immune cells infiltrating the brain, and their peripheral variations have been recently reviewed [26], even if studied mostly in animal models. Neutrophils, monocytes, and lymphocytes increased in the ischemic hemisphere in just a few hours, and could be found locally for 15–30 days after the ischemic event. Peripherally, after an ischemic stroke, there is an exponential increase in the neutrophil count together with a decrease in the lymphocyte count [27]. What happens in the long-lasting phase after the stroke is less known. In a retrospective study, in stroke patients treated with recombinant tissue plasminogen activator, increased numbers of circulating monocytes were associated with a poorer outcome [28]. In the Mugello study, the localization and the dimension of the ischemic lesion, and the age at the stroke event, were not assessed, but we might surmise that those robust oldest-old survived to stroke thanks to a low monocyte absolute count.

To the best of our knowledge, this is the first report describing the association between lymphocyte-to-monocyte ratio and dementia in a cohort of oldest-old. The probable/possible relative increase of the adaptive, simultaneous with a probable/possible relative reduction of the innate, immune response, is amenable to different hypotheses. For instance, it may represent an example of adaptative/maladaptative immunological response in the pathogenesis of dementia [29]. The overstimulated immune system, activated perhaps by micro-bleeding and/or Aβ plaque formation, may lead to an “immune paralysis” state [30]. Ultimately, the immune system cannot generate an adequate cellular response [29], with increased immunosuppressive activity on inflammation, targeted to protect neurons, but de facto inducing an increase of Aβ plaque deposition [7]. Alternatively, the imbalance between innate/adaptive immunity in this cohort of nonagenarians may just be due to the aging leukocytes’ endowment (immunobiography), to be considered independently from dementia [31]. Lastly, the Mugello study enrolled a “successful and robust” cohort of nonagenarians, since they were long-survivors. Probably, as for centenarians, they may show reduced inflamm-aging and slower immunosenescence that enable them to reach this extreme phase of life [32]. This hypothesis could explain why subjects with a diagnosis of dementia showed a less inflamed phenotype. Indeed, it is difficult to suggest the leukocytes ratios as helpful in the early diagnosis of dementia, since they may reflect the “later immunological clinical phase” of dementia, with a reduction in adaptive immune activity [4].

Two of the most important factors (hypertension and overweight) classically associated with aging, inflammation, and cardiovascular events apparently do not affect the association between leukocytes ratio and dementia. Interestingly, higher lymphocyte counts are associated with a clinical diagnosis of dementia. Since the analysis is adjusted for age and sex, this association cannot be justified by chronological age or sex dimorphism [33]. In the multivariate models, the differences in lymphocyte numbers between the two groups were no longer present. We did not find lymphopenia to be a common feature in our sample. This is at variance with the reported lymphopenia in degenerative dementia, or with the similar lymphocyte counts observed in Alzheimer’s disease patients and controls [34]. However, our data refer to a free-living population of nonagenarians, which may result in a selection of dementia cases.

## Strengths and limitations

The present study has several strengths and limitations that deserve comments. The Mugello study offers an opportunity to investigate the association between blood peripheral cell-count ratios and clinical diagnosis of dementia, in a cohort of oldest-old representative of the Italian general population. Since leukocyte-ratios integrate information from different cell types, they have higher informative content than single inflammatory biomarkers, and their use is potentially advantageous [35]. Moreover, the relationship between LMR and cognitive status in nonagenarians has not been previously investigated.

As a limitation, our cross-sectional study naturally precludes the possibility to establish a causal relationship of the associations, and to assign a prognostic value to LMR in dementia progression. As a further limitation, the diagnosis of dementia in our study is clinical, and retrospective; thus, no attempt was made to classify the subtype or disease stage. Furthermore, subjects in the asymptomatic stage or affected by mild cognitive impairment were not identified. Results reported in this study do not uniquely support an inflammatory paradigm for the development of dementia, since the association of leukocyte-derived ratios might reflect a subclinical response to infections, which are a highly probable and frequent event in frail and compromised subjects [36]. Finally, several confounding factors, such as the ApoE genotype, significantly affecting dementia risk, were not explored in this study.

## Conclusion

In a population of free-living nonagenarians, higher LMR was associated with late-life any type dementia. However, this association was no longer observed in participants with dementia who had a previous stroke. The hypothesis that LMR is associated to any type dementia must be further verified. Since LMR is a low-cost and easily accessible index, longitudinal studies of its use as a biomarker for the diagnosis of neurodegenerative dementia in different stages should be planned.

## Data Availability

The Mugello study dataset is not stored in a data repository, but data are available on reasonable request.
